# Association between tinnitus and depressive symptoms in the South Korean population

**DOI:** 10.1371/journal.pone.0261257

**Published:** 2021-12-20

**Authors:** Minah Park, Soo Hyun Kang, Fatima Nari, Eun-Cheol Park, Sung-In Jang

**Affiliations:** 1 Department of Public Health, Graduate School, Yonsei University, Seoul, South Korea; 2 Institute of Health Services Research, Yonsei University, Seoul, South Korea; 3 Department of Preventive Medicine, Yonsei University College of Medicine, Seoul, South Korea; Gachon University Gil Medical Center, REPUBLIC OF KOREA

## Abstract

In this study, we aimed to examine the association between tinnitus and depressive symptoms in middle-aged and elderly South Korean population. The participants were selected from among those who participated in the 2014, 2016, and 2018 Korea National Health and Nutrition Examination Surveys. The incidence and severity of tinnitus was assessed using a self-reported questionnaire, while depressive symptoms were evaluated using self-reported Patient Health Questionnaire-9. Multiple logistic regression was performed to examine the association between tinnitus and depressive symptoms. Overall, 10 979 (4821 men and 6158 women) participants were enrolled in the study. Regardless of sex, individuals who reported having tinnitus were more likely to have depressive symptoms than those without tinnitus (men: odds ratio 1.53, 95% confidence interval 1.01–2.32; women: odds ratio 1.78, 95% confidence interval 1.35–2.35). In severe cases of tinnitus, women were more likely to have depressive symptoms (odds ratio 7.18, 95% confidence interval 3.71–13.87) compared to men. This study revealed a significant association between tinnitus and depressive symptoms among the middle-aged and elderly South Korean population.

## Introduction

Tinnitus is a condition of noise perception in which symptoms include hearing sounds such as buzzing, roaring, and hissing, among others. It is a common problem affecting an average of 15–20% of the population in the United States [1]. More than 95% of people experience tinnitus at least once in their lives. Individuals may develop tinnitus due to various reasons; however, tinnitus due to either central nervous system issues or emotional distress can be particularly challenging to treat [2]. In South Korea, the number of people who develop tinnitus has increased over time, from 19.7% in 2010 to 21.4% in 2012 [3]. Recently, the Korean National Health Insurance reported that the number of patients with tinnitus increased by 3% annually, and the medical care associated with this condition increased annually by 5.6%, which amounted to a medical burden of 22.7 billion KRW in 2013 [4].

Depression is a common mental disorder that affects 265 million people globally across all ages. It was among the top five causes for years lived with disability in 2017 [5]. Depression may not only affect various aspects of life such as family, work, and school, but at its worst, may lead to suicide [6]. In 2015, South Korea recorded the highest suicide rate among the Organization for Economic Co-operation and Development countries, which was 2.4 times higher than the average suicide rate [7]. Over the years, mental health problems have become a national issue in South Korea. The Korean Epidemiologic Catchment Area study, an epidemiological survey on mental health conducted every five years, has reported a gradual increase in the prevalence of depression in the recent years (2001, 4.0%; 2006, 5.6%; 2011, 6.7%) [8].

Among patients who experience tinnitus, some learn to cope with the phantom perception, while for others, the condition can be more difficult [9]. Patients with tinnitus consider it a critical nuisance, which has a negative impact on their life and can lead to depression, or in extreme cases, suicide [10]. With the rise in the elderly population, a consequent increase in the incidence of both depression and tinnitus may be observed.

Although other studies [11–15] have observed a relationship between tinnitus and depression in different population groups, most studies perceived depression as a single response question. However, no previous study in South Korea has evaluated patients with tinnitus using the Patient Health Questionnaire 9 (PHQ-9). Use of the PHQ-9 in clinical settings is well-known, and it can be used to assess the severity of depression. Given that depression is a common mental illness and is widely prevalent in the population, it warrants the need for a better evaluation of a nationwide representative sample.

Therefore, the purpose of this study was to analyze the association between tinnitus and depressive symptoms using nationally representative data. Additionally, we aimed to examine this association stratified by sex.

## Materials and methods

### Data

The data used in this study were obtained from the Korea National Health and Nutrition Examination Survey (KNHANES) conducted in 2014, 2016, and 2018 by the Korea Center for Disease Control and Prevention Agency. The KNHANES is a nationwide survey conducted annually to evaluate health behavior and health status, with slight modifications made each year in the parameters being assessed. The data from these surveys are used to monitor and estimate the prevalence of chronic diseases in the South Korean population. The KHANES sampling plan has a multistage clustered probability design. For this study, 192 primary sampling units (PSUs) were drawn from ~200 000 geographically defined PSUs for the entire country. A PSU comprises an average of ~60 households; among these, 20 households were chosen for each PSU using a systemic sampling method [16]. The study was reviewed by the Institutional Review Board of the Korea Centers for Disease Control and Prevention and was conducted according to the principles of the Declaration of Helsinki. All participants signed an informed consent form [17].

### Participants

Among the 23,692 participants, characteristics of those excluded from the study were as follows: participants younger than 40 years (10,075 participants), those previously diagnosed with depression (819 participants), those who failed to complete the PHQ-9 questions (1,575 participants), those who failed or refused to answer regarding tinnitus (112 participants), and those who reported incomplete data regarding education level, region, marital status, occupation, household income, smoking habits, alcohol use, subjective health condition, and stress (132 participants). In total, 10,979 participants (men, 4,821; women, 6,158) were included in the study. In the years 2014, 2016, and 2018, KNHANES only surveyed participants aged ≥ 40 years for tinnitus.

### Variables

The main variable of interest in this study was tinnitus status. In the KNHANES, each individual was asked, “Have you heard any ringing, buzzing, roaring, or hissing sounds without an external acoustic source in the past year?” with the response option of “Yes” or “No” [11]. In the follow-up question, each individual was asked, “How much did the sound originating in your ear disturb your daily life?” with the response options of “Not bothersome”, “Bothersome and annoying”, and “Bothersome enough to be unable to sleep”. These were then reclassified with “none” as no tinnitus, “mild” as “not bothersome”, “moderate” as “bothersome and annoying”, and “severe” as “bothersome enough to be unable to sleep”.

The dependent variable was the PHQ-9 score. The PHQ-9 is a self-reported questionnaire for screening depression and includes nine questions. Each question is rated as 0 (“not at all”), 1 (“for many days”), 2 (“for more than a week”), and 3 (“almost daily”), resulting in a maximum score of 27 points [18]. It has been proven to be a reliable tool not only for screening individuals with depression but also for evaluating the severity of the illness [19]. Individuals were considered to show depressive symptoms if they scored > 10 points on this questionnaire [20].

We controlled for covariates such as sociodemographic and socioeconomic factors, health behaviors, and health conditions of the participants in this study. The sociodemographic factors were age and sex. The socioeconomic factors were education level, region, marital status, occupation, and household income. Occupation was categorized as white (managerial, professional, or clerical), pink (services or sales), blue (manual labor), and unemployed. Health behaviors included smoking habits and alcohol use. Health conditions included subjective health condition and stress.

### Statistical analyses

A descriptive analysis was performed to examine the distribution of the general characteristics of the study population. Multiple logistic regression analysis was performed to evaluate the relationship between tinnitus and depression after accounting for potential confounding variables including demographic, socioeconomic, and health-related characteristics. The results were reported using odds ratios (ORs) and 95% confidence intervals (CIs). Model fitting was performed using the PROC SURVEYLOGISTIC procedure and application of weight procedures, cluster, and strata. All analyses were conducted using SAS 9.4 (SAS Institute Inc; Cary, North Caroline). A P-value <0.05 was considered statistically significant.

## Results

Table 1 presents the results of the general characteristics. Among the 10 979 participants, 4821 were men and 6158 were women. The overall incidence rate of depressive symptoms was higher in women than in men (5.7% vs. 3.2%). For the participants who reported tinnitus, the incidence rate in women was twice that in men (10.3% vs. 5.0%) (P<0.001).

**Table 1:** General characteristics of the study population.

Variables	PHQ-9≥10 (Depression)									
	Men (n = 4,821)					Women (n = 6,158)				
	Yes		No			Yes		No		
	N	%	N	%	P Value	N	%	N	%	P Value
**Total(n = 10,979)**	**153**	**(3.2)**	**4667**	**(96.8)**		**351**	**(5.7)**	**5,807**	**(94.3)**	
**Tinnitus**					< .0001					< .0001
Yes	57	(5.0)	1,084	(95.0)		152	(10.3)	1,320	(89.7)	
No	96	(2.6)	3,584	(97.4)		199	(4.2)	4,487	(95.8)	
**Age**					0.2693					< .0001
40 ~ 49	33	(2.7)	1,203	(97.3)		52	(3.2)	1,559	(96.8)	
50 ~ 59	34	(2.8)	1,191	(97.2)		72	(4.2)	1,626	(95.8)	
60 ~ 69	40	(3.2)	1,191	(96.8)		96	(6.7)	1,347	(93.3)	
70 ~ 79	35	(3.9)	866	(96.1)		98	(9.1)	975	(90.9)	
80 ~	11	(4.8)	217	(95.2)		33	(9.9)	300	(90.1)	
**Region**					0.5631					0.4086
Metropolitans	77	(3.0)	2,460	(97.0)		181	(5.5)	3,126	(94.5)	
Rurals	76	(3.3)	2,208	(96.7)		170	(6.0)	2,681	(94.0)	
**Educational level**					< .0001					< .0001
Under Middle School	81	(4.8)	1,591	(95.2)		254	(8.6)	2,707	(91.4)	
High School	37	(2.4)	1,486	(97.6)		69	(3.7)	1,788	(96.3)	
University or Higher	35	(2.2)	1,591	(97.8)		28	(2.1)	1,312	(97.9)	
**Occupation**					< .0001					< .0001
White	21	(1.8)	1,170	(98.2)		14	(1.7)	833	(98.3)	
Pink	10	(2.4)	414	(97.6)		49	(4.7)	987	(95.3)	
Blue	39	(2.1)	1,817	(97.9)		59	(4.9)	1,136	(95.1)	
Unemployed	83	(6.1)	1,267	(93.9)		229	(7.4)	2,851	(92.6)	
**Household Income**					< .0001					< .0001
High	15	(1.1)	1,352	(98.9)		26	(1.6)	1,584	(98.4)	
Middle high	21	(1.6)	1,280	(98.4)		61	(4.0)	1,456	(96.0)	
Middle low	40	(3.4)	1,151	(96.6)		92	(6.0)	1,448	(94.0)	
Low	77	(8.0)	885	(92.0)		172	(11.5)	1,319	(88.5)	
**Marital Status**					0.0045					0.2089
Yes	139	(3.0)	4,466	(97.0)		341	(5.6)	5,697	(94.4)	
No	14	(6.5)	202	(93.5)		10	(8.3)	110	(91.7)	
**Subjective Health Condition**					< .0001					< .0001
Good	7	(0.5)	1,460	(99.5)		16	(1.1)	1,497	(98.9)	
Normal	41	(1.6)	2,458	(98.4)		108	(3.3)	3,142	(96.7)	
Bad	105	(12.3)	750	(87.7)		227	(16.3)	1,168	(83.7)	
**Smoking Habit**					0.0016					< .0001
Yes	69	(4.3)	1,536	(95.7)		36	(14.5)	213	(85.5)	
No	84	(2.6)	3,132	(97.4)		315	(5.3)	5,594	(94.7)	
**Alcohol Use**					0.8264					0.0206
Yes	104	(3.1)	3,212	(96.9)		100	(4.8)	2,005	(95.2)	
No	49	(3.3)	1,456	(96.7)		251	(6.2)	3,802	(93.8)	
**Stress**					< .0001					< .0001
Yes	144	(3.9)	3,567	(96.1)		331	(6.8)	4,513	(93.2)	
No	9	(0.8)	1,101	(99.2)		20	(1.5)	1,294	(98.5)	

Table 2 presents the general characteristics of participants with tinnitus. Overall, among people with tinnitus were more likely to be unemployed, have low household income and have bad subjective health condition.

**Table 2:** Demographic characteristics of the study population according to tinnitus.

Variables	Tinnitus									
	Men (n = 4,821)					Women(n = 6,158)				
	Yes		No			Yes		No		
	N	%	N	%	P Value	N	%	N	%	P Value
**Total(n = 10,979)**	**1,141**	**(23.7)**	**3,680**	**(76.3)**		**1,472**	**(23.9)**	**4,686**	**(76.1)**	
**Age**					< .0001					< .0001
40 ~ 49	218	(17.6)	1,018	(82.4)		308	(19.1)	1,303	(80.9)	
50 ~ 59	256	(20.9)	969	(79.1)		341	(20.1)	1,357	(79.9)	
60 ~ 69	321	(26.1)	910	(73.9)		372	(25.8)	1,071	(74.2)	
70 ~ 79	281	(31.2)	620	(68.8)		326	(30.4)	747	(69.6)	
80 ~	65	(28.5)	163	(71.5)		125	(37.5)	208	(62.5)	
**Region**					0.2646					0.0334
Metropolitans	584	(23.0)	1,953	(77.0)		755	(22.8)	2,552	(77.2)	
Rurals	557	(24.4)	1,727	(75.6)		717	(25.1)	2,134	(74.9)	
**Educational level**					< .0001					< .0001
Under Middle School	489	(29.2)	1,183	(70.8)		872	(29.4)	2,089	(70.6)	
High School	336	(22.1)	1,187	(77.9)		363	(19.5)	1,494	(80.5)	
University or Higher	316	(19.4)	1,310	(80.6)		237	(17.7)	1,103	(82.3)	
**Occupation**					< .0001					< .0001
White	203	(17.0)	988	(83.0)		144	(17.0)	703	(83.0)	
Pink	87	(20.5)	337	(79.5)		196	(18.9)	840	(81.1)	
Blue	448	(24.1)	1,408	(75.9)		287	(24.0)	908	(76.0)	
Unemployed	403	(29.9)	947	(70.1)		845	(27.4)	2,235	(72.6)	
**Household Income**					< .0001					< .0001
High	279	(20.4)	1,088	(79.6)		314	(19.5)	1,296	(80.5)	
Middle high	261	(20.1)	1,040	(79.9)		309	(20.4)	1,208	(79.6)	
Middle low	295	(24.8)	896	(75.2)		379	(24.6)	1,161	(75.4)	
Low	306	(31.8)	656	(68.2)		470	(31.5)	1,021	(68.5)	
**Marital Status**					0.4242					0.7158
Yes	1,085	(23.6)	3,520	(76.4)		1,445	(23.9)	4,593	(76.1)	
No	56	(25.9)	160	(74.1)		27	(22.5)	93	(77.5)	
**Subjective Health Condition**					< .0001					< .0001
Good	261	(17.8)	1,206	(82.2)		236	(15.6)	1,277	(84.4)	
Normal	593	(23.7)	1,906	(76.3)		747	(23.0)	2,503	(77.0)	
Bad	287	(33.6)	568	(66.4)		489	(35.1)	906	(64.9)	
**Smoking Habit**					0.8936					0.4968
Yes	378	(23.6)	1,227	(76.4)		64	(25.7)	185	(74.3)	
No	763	(23.7)	2,453	(76.3)		1,408	(23.8)	4,501	(76.2)	
**Alcohol Use**					0.0036					< .0001
Yes	745	(22.5)	2,571	(77.5)		428	(20.3)	1,677	(79.7)	
No	396	(26.3)	1,109	(73.7)		1,044	(25.8)	3,009	(74.2)	
**Stress**					< .0001					0.0001
Yes	888	(23.9)	2,823	(76.1)		1,210	(25.0)	3,634	(75.0)	
No	253	(22.8)	857	(77.2)		262	(19.9)	1,052	(80.1)	

Table 3 reports the findings of the logistic regression analysis stratified by sex for the association between depressive symptoms and tinnitus for all variables. Regardless of sex, participants who developed tinnitus had increased odds of developing depressive symptoms (women: OR 1.78, CI 1.35–2.35; men: OR: 1.53, CI 1.01–2.32).

**Table 3:** Associations between depressive symptoms (PHQ-9) and subject demographics.

Variables	Depressive symptoms (PHQ-9* ≥10)							
	Men				Women			
	OR	95% CI			OR	95% CI		
**Tinnitus**								
Yes	1.53	(1.01	-	2.32)	1.78	(1.35	-	2.35)
No	1.00				1.00			
**Age**								
40 ~ 49	1.00				1.00			
50 ~ 59	0.69	(0.38	-	1.25)	0.96	(0.64	-	1.44)
60 ~ 69	0.55	(0.28	-	1.08)	0.99	(0.63	-	1.56)
70 ~ 79	0.41	(0.18	-	0.89)	1.07	(0.64	-	1.80)
80 ~	0.49	(0.17	-	1.42)	1.26	(0.69	-	2.32)
**Region**								
Metropolitans	1.00				1.00			
Rurals	1.00	(0.67	-	1.49)	0.80	(0.61	-	1.03)
**Educational level**								
Under Middle School	0.87	(0.47	-	1.60)	1.43	(0.84	-	2.44)
High School	0.78	(0.41	-	1.46)	1.20	(0.69	-	2.10)
University or Higher	1.00				1.00			
**Occupation**								
White	1.00				1.00			
Pink	1.24	(0.53	-	2.92)	1.53	(0.72	-	3.23)
Blue	0.77	(0.39	-	1.53)	1.34	(0.64	-	2.79)
Unemployed	2.40	(1.09	-	5.26)	1.69	(0.86	-	3.34)
**Household income**								
High	1.00				1.00			
Middle high	1.09	(0.50	-	2.33)	1.64	(0.93	-	2.90)
Middle low	2.38	(1.11	-	5.11)	1.91	(1.11	-	3.29)
Low	4.40	(1.98	-	9.76)	2.90	(1.69	-	4.96)
**Marital Status**								
Yes	1.00				1.00			
No	1.07	(0.51	-	2.23)	1.23	(0.58	-	2.59)
**Subjective Health Condition**								
Good	1.00				1.00			
Normal	2.02	(0.80	-	5.07)	2.53	(1.40	-	4.58)
Bad	13.00	(5.22	-	32.36)	10.58	(5.96	-	18.80)
**Smoking Habit**								
Yes	1.63	(1.06	-	2.51)	4.84	(2.88	-	8.15)
No	1.00				1.00			
**Alcohol Use**								
Yes	1.22	(0.74	-	1.99)	0.86	(0.64	-	1.17)
No	1.00				1.00			
**Stress**								
Yes	6.21	(2.81	-	13.72)	4.83	(3.08	-	7.57)
No	1.00				1.00			

Table 4 reports the findings of the subgroup analysis of 497 participants, stratified by the independent variables after adjusting for multiple comparison. Men participants in the age range of 40–49 years (OR 2.96; CI 1.30–6.72) had a higher risk of depressive symptoms. Women participants in the age range of 70–79 years (OR 2.00; CI 1.14–3.53), regardless of alcohol consumption, had a higher odds of risk of depressive symptoms.

**Table 4:** Subgroup analysis stratified by independent variables.

Variables	PHQ-9 ≥10									
	Tinnitus									
		Men				Women				
	No	Yes				No	Yes			
	OR	OR	95% CI			OR	OR	95% CI		
**Age**										
40–49	1.00	2.96	(1.30	-	6.72)	1.00	1.97	(0.92	-	4.23)
50–59	1.00	1.09	(0.45	-	2.63)	1.00	1.50	(0.85	-	2.66)
60–69	1.00	1.08	(0.48	-	2.43)	1.00	1.26	(0.73	-	2.17)
70–79	1.00	1.23	(0.54	-	2.80)	1.00	2.00	(1.14	-	3.53)
80~	1.00	3.42	(0.32	-	36.18)	1.00	2.40	(0.96	-	5.99)
**Occupation**										
White	1.00	1.72	(0.51	-	5.80)	1.00	4.08	(1.32	-	12.65)
Pink	1.00	6.63	(1.12	-	39.27)	1.00	1.53	(0.65	-	3.60)
Blue	1.00	1.59	(0.68	-	3.74)	1.00	1.98	(1.04	-	3.76)
Unemployed	1.00	1.10	(0.60	-	2.03)	1.00	1.60	(1.12	-	2.29)
**Region**										
Metropolitan area	1.00	1.17	(0.62	-	2.22)	1.00	2.03	(1.37	-	3.01)
Rural	1.00	1.98	(1.13	-	3.46)	1.00	1.48	(1.00	-	2.19)
**Educational level**										
Middle school or less	1.00	1.49	(0.84	-	2.64)	1.00	1.69	(1.22	-	2.35)
High school	1.00	2.28	(1.02	-	5.08)	1.00	1.38	(0.76	-	2.51)
College or over	1.00	1.61	(0.59	-	4.34)	1.00	3.27	(1.37	-	7.79)
**Alcohol consumption**										
Yes	1.00	1.33	(0.81	-	2.20)	1.00	1.95	(1.12	-	3.39)
No	1.00	1.79	(0.82	-	3.90)	1.00	1.73	(1.25	-	2.41)
**Income**										
High	1.00	1.21	(0.27	-	5.44)	1.00	4.52	(1.79	-	11.45)
Middle high	1.00	0.74	(0.23	-	2.30)	1.00	1.19	(0.61	-	2.31)
Middle low	1.00	2.25	(0.99	-	5.12)	1.00	1.12	(0.63	-	2.00)
Low	1.00	1.52	(0.83	-	2.76)	1.00	2.29	(1.51	-	3.45)

**Fig 1:**
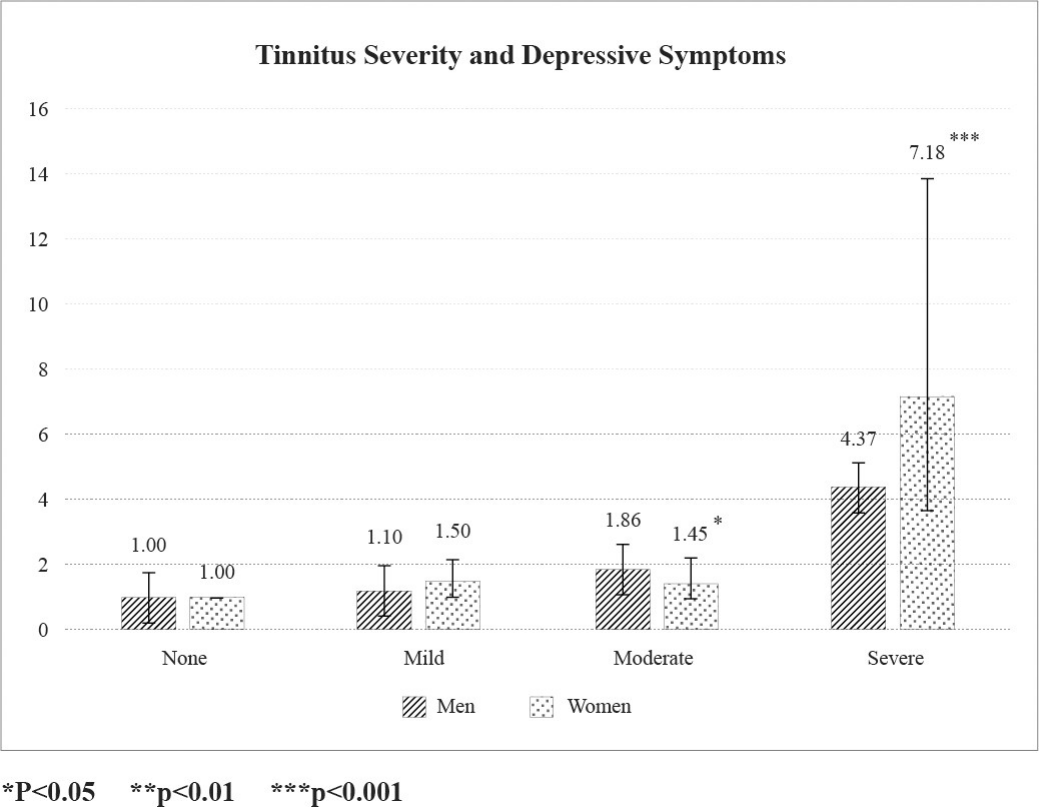
Association between tinnitus severity and depressive symptoms, stratified by sex.

Fig 1 presents the results for tinnitus severity and depressive symptoms reported by the participants. Women participants with the most severe form of tinnitus had higher odds of risk of depression (OR 7.18; CI 3.71–13.87; P<0.001) compared to men.

## Discussion

In this study, we examined the association between tinnitus and depression among the South Korean Population. This study revealed a significant correlation between the incidence of tinnitus and depression in both men and women. It also revealed that, compared to those with no tinnitus, the more severe the tinnitus was, the more the likelihood of depressive symptoms.

Our findings, along with other previous studies, present a large base of evidence for the relationship between tinnitus and depression [21]. Although the exact mechanism underlying the relationship is not known, the predominant theory is that among depression-prone individuals, tinnitus sets off depression [22]. Another theory is that psychological processes contribute to the deterioration and severity of the tinnitus [23].

This study revealed a significant correlation between the incidence of tinnitus and depression in both men and women. According to our analysis, 40- to 49-year-old men with tinnitus were more likely to have depressive symptoms, which may have been due to the combination of factors related to the age group and work culture. In South Korea, 40–49 years is the age range during which men may face early retirement, along with the social pressure and consequences of not being able to work and support their families until their actual retirement age [24]. According to the Hearing Health Foundation, even in individuals with a typical working environment, tinnitus can cause pain or spikes in the ear, causing them to leave their jobs [25]. Among men, professional work is highly linked to the perception of one’s masculinity; hence, failure to execute professional activities and duties may have stronger psychological effects on them, resulting in a higher likelihood of depression [26].

In contrast, women with tinnitus showed an increased likelihood of exhibiting depressive symptoms in their 70s. This might have been due to the fact that hearing loss, a risk factor for tinnitus, is more prevalent in older age. In the United States, one in three people between the ages 65 and 74 years reports hearing loss, and nearly half of the population aged >75 years has difficulty in hearing [27]. For senior citizens, good health in general means an independent and highly content life. However, since most elderly people develop age-related health issues, they are more likely to develop depressive symptoms [28].

This study also revealed that women were likely to have depressive symptoms, while men were not likely to have depressive symptoms regardless of their alcohol consumption status. A previous study reported an inverse relationship between alcohol consumption and tinnitus, stating that alcohol has a protective effect on the microvascular health of the cochlea [11]. However, in another study, no association was observed between alcohol consumption and tinnitus [29]. As for our study, we were unable to find an association between alcohol consumption, tinnitus, and depressive symptoms.

Further, regardless of the tinnitus severity, women were more likely to have depressive symptoms than men in our study. This could be due to the differences in coping with stress. A previous study reported that women had a higher vulnerability to repeated stress and struggled with the habituation process of tinnitus [30], which is in line with the findings reported by Stouffer et al. [31]. Moreover, due to more culture-based perceptions in South Korea, women are expected to be obedient and fulfil more roles in the family. This might result in women being more prone to stress [32]. Japan, which has a culture similar to that of South Korea, also showed a higher prevalence of tinnitus among women than among men [33].

This study has some limitations. First, it was based on data from a cross-sectional study. Therefore, although the association could be confirmed, the causality could not be evaluated. Second, the KNHANES only collected tinnitus-related data from patients aged ≥40 years. Due to the change in lifestyles, including the use of headphones [34] and mandatory military service for men in their 20s [35], people in the age range of 20–40 years have been reported to have an increased risk of tinnitus. Thus, future studies should include a younger population. Third, there were no standardized questionnaires on tinnitus. Fourth, we could not obtain any information regarding hearing loss, somatic symptoms, and other psychopathologic symptoms. Despite these limitations, this study has several strengths. We used the most recent nationally representative database to determine the association between tinnitus and depressive symptoms. Therefore, the results obtained are highly representative of the middle-aged and older population of South Korea. In addition, we utilized the self-reported PHQ-9, an easy and valid tool for screening patients with depressive symptoms. To the best of our knowledge, this is the first study to determine the association between tinnitus and depressive symptoms using the PHQ-9 with KNHANES data.

## Conclusion

In conclusion, this study revealed a significant correlation between tinnitus and depressive symptoms in the middle-aged and elderly South Korean population. Furthermore, regardless of the severity of tinnitus, women were more likely to have depressive symptoms than men.

## Supporting information

S1 Table Association between tinnitus severity and depression. (DOCX)
