# Supplementary material for: Association between tinnitus and depressive symptoms in the South Korean population
Source: PLoS One. 2021 Dec 20;16(12):e0261257. doi: 10.1371/journal.pone.0261257 (PMC8687527; doi:10.1371/journal.pone.0261257)
Supplement: S1 Table — (DOCX) [file pone.0261257.s001.docx]

**S1 Table.** **Association between Tinnitus Severity and Depression**

|  | | |  |  |  |
| --- | --- | --- | --- | --- | --- |
| **Variables** |  | **Depression (PHQ-9 ≥10)** | | | |
|  |  | **Tinnitus** | | | |
|  | **N** | **OR** | **95% CI** | | |
| **Men** |  |  |  |  |  |
| None | 3680 | 1.00 |  |  |  |
| Mild | 45 | 1.19 | (0.66 | - | 2.12) |
| Moderate | 384 | 1.86 | (1.08 | - | 3.19) |
| Severe | 45 | 4.37 | (0.98 | - | 19.34) |
| **Women** |  |  |  |  |  |
| None | 4686 | 1.00 |  |  |  |
| Mild | 855 | 1.50 | (1.06 | - | 2.12) |
| Moderate | 525 | 1.45 | (1.00 | - | 2.10) |
| Severe | 92 | 7.18 | (3.71 | - | 13.87) |
